# Rat models for low and high adaptive response to exercise differ for stress‐related memory and anxiety

**DOI:** 10.14814/phy2.14716

**Published:** 2021-02-22

**Authors:** William M. Vanderheyden, Michaela Kehoe, Giancarlo Vanini, Steven L. Britton, Lauren Gerard Koch

**Affiliations:** ^1^ Department of Biomedical Sciences Washington State University Spokane WA USA; ^2^ Department of Anesthesiology University of Michigan Ann Arbor MI USA; ^3^ Department of Physiology and Pharmacology The University of Toledo College of Medicine and Life Sciences Toledo OH USA

**Keywords:** aerobic fitness, cognition, exercise, neurobiology, rat models, response to training, stress

## Abstract

Physical exercise and fitness may serve as resilience factors to stress exposure. However, the extreme range in human exercise performance suggests that genetic variation for exercise capacity could be a confounding feature to understanding the connection between exercise and stress exposure. To test this idea, we use laboratory rat models selectively bred for a low and high gain in aerobic running capacity in response to training to examine whether an inherent capacity to respond to physical exercise reflects how stress changes neurobiological functioning and regulates fear‐associated memory processing. Utilization of this contrasting rat model system of low and high responders has the potential to guide the interpretation of the reported association with exercise involvement and the reduction of stress‐induced anxiety disorders. Our data show that aerobic fitness may be linked to the ability to regulate fear‐associated memories. We also show that acquired exercise capacity may play a key role in regulating responses to an acute stressor. Exercise sensitivity plays a significant role in the activation of the plasticity‐associated molecule extracellular signal‐regulated kinase, changes in stress hormone activity, and anatomical modifications to the noradrenergic locus coeruleus. These data identify a unique operational mechanism that may serve as translational targets for lessening symptoms of stress and anxiety.

## Graphical abstract

Rats selectively bred for low and high response to physical exercise training show a differential ability to extinguish or recall fear‐associated memories. Exercise sensitivity plays a significant role in activation of the plasticity‐associated molecule extracellular signal‐regulated kinase, changes in stress hormone activity, and anatomical modifications to the noradrenergic locus coeruleus. These data identify a unique operational mechanism that may serve as translational targets for lessening symptoms of stress and anxiety.

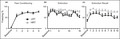

**Highlights**

- Rats selectively bred for low and high response to physical exercise training show a differential ability to extinguish or recall fear‐associated memories.
- High and low responders to exercise training show the differential activation of the stress hormone system.
- Plasticity‐associated molecules are differentially regulated by stress in these selectively bred animals.
- Rats bred to be more sensitive to exercise training results in structural anomalies in the noradrenergic locus coeruleus.

## 1 INTRODUCTION

Physical and psychological stress can cause long‐term changes in neurobiological circuits (Bremner et al., 2007) and impair the extinction of fear‐associated memories (Garfinkel et al., 2014). Stress impairs the function of the stress hormone system (Baker et al., 1999; Yehuda et al., 2004), leads to epigenetic alterations (Zovkic & Sweatt, 2013), results in impaired learning and memory neurobiology (Martini et al., 2013), and causes inflammatory response pathways to become activated (Maes et al., 1999; Spivak et al., 1997).

Similarly, physical exercise training has also been shown to increase oxidative stress (Dillard et al., 1978), lead to epigenetic changes (Chandramohan et al., 2008; Denham et al., 2014), and to regulate stress hormones (Freund et al., 1991; Kloet et al., 2005). However, in contrast to physical or psychological stress, exercise is generally accepted as being healthy for the mind and body by increasing neurogenesis (Praag et al., 1999) and improving learning and memory (Ahmadiasl et al., 2003). In fact, exercise may be a useful therapy for anxiety and stress disorders (Barbour et al., 2007; Blumenthal et al., 2007).

Physical fitness is hypothesized to be a resilience factor in the acquisition of stress‐related disorders (Taylor et al., 2008; Zen et al., 2012). However, the neural and molecular mechanisms leading to this type of resilience are poorly understood. To assess the molecular and anatomical regulation of fear‐associated memory processing, we examined the response to fear conditioning, fear extinction, and fear extinction recall in strains of rats that differ in their adaptive response to aerobic treadmill training. We developed high response trainer (HRT) and low response trainer (LRT) rats by divergent selective breeding for gain in exercise capacity after performing the same amount of treadmill training (Koch et al., 2013). Compared to HRT rats, LRT rats fail to increase VO_2max_, show blunted cardiac muscle remodeling response, have altered signal transduction for JNK and p38 mitogen‐activated protein kinase, reduced mitochondrial biogenesis regulating factors in skeletal muscle (PGC1‐α, NRF1, and TFAM), and lower hippocampal neurogenesis in response to aerobic exercise training suggesting impaired plasticity (Lessard et al., 2013; Marton et al., 2014; Nokia et al., 2016; Wisloff et al., 2015). It also appears that exercise training differently modified the results on a passive avoidance test used to evaluate learning and memory. The HRT rats had a decline in recall after gaining an improvement in exercise capacity whereas the LRT rats were not altered (Marton et al., 2016). Thus, the motivation for this study was to discover whether this animal model system of contrasting sensitivity to exercise training may be uniquely suited for studying the genotype‐to‐phenotype connection between physical exercise, stress, and the processing of fear‐associated memories.

In addition to behavioral responses to fear conditioning, we also examined the neuro‐anatomical regulation of plasticity‐related molecules in response to an acute 2‐hour physical restraint stressor in LRT and HRT animals. Extracellular signal‐regulated kinase (ERK), glucocorticoid receptor (GR), tyrosine hydroxylase (TH), and brain‐derived neurotrophic factor (BDNF) have been identified to be regulated by both exercise (Holmes, 2014; Rothman & Mattson, 2013; Widegren et al., 2001) and stress (Sciolino & Holmes, 2012; Takei et al., 2011). We performed these experiments in order to assess whether key molecular contributions to the fear memory processing centers of the brain differ between LRT and HRT rats. We chose to assess changes in brain areas previously identified to be contributing to aberrant stress responses and fear memory processing abnormalities (reviewed in Liberzon & Sripada, 2008; Pitman et al., 2012; Sripada et al., 2012; Vanderheyden et al., 2014).

We hypothesize that inherited variation for aerobic gain in response to physical training may play a role in mediating plasticity‐related responses that affect stress, memory, and anxiety pathways. Due to the differential activation of plasticity‐related molecules and their importance in mediating gene transcription, the data herein suggest that fear‐associated memory impairments may be due to altered epigenetic mechanisms in a circuit dependent manner or by structural changes in the noradrenergic system. Further experimentation on these animals, however, is required to determine the specific epigenetic mechanisms mediating the interaction between physical fitness and stress exposure. Last, we show that the HRT and LRT rats are a unique animal model system that can be used to understand the relationships between the processing of fear‐associated memories, and the molecular and anatomical interactions of stress and physical exercise.

## 2 METHODS

### 2.1 General animal procedures

All animal procedures were carried out in accordance with the National Institutes of Health Guide for the Care and Use of Laboratory Animals and with approval from the University of Michigan Committee on the Use and Care of Laboratory Animals (UCUCA). The LRT and HRT selected lines are developed by Koch and Britton and currently maintained in a specific pathogen‐free facility at the University of Toledo (Koch et al., 2013). Starting with a founder population of genetically heterogeneous rats (N/NIH stock), the two‐way artificial selection was performed across several generations based on the magnitude of change in running capacity after completing 8 weeks of standardized aerobic treadmill training. Animals are phenotyped for the response to training starting at 3 months of age and completed by 6 months of age. For this study, a cohort of 20‐month‐old males from generation 20 of selection was housed individually in sound‐attenuating boxes on a 12:12 hr, light:dark schedule at constant temperature (23°C) and humidity (20%) and given ad libitum access to rat chow and water.

### 2.2 Fear conditioning, fear extinction, and extinction recall

Fear conditioning experiments were conducted using eight High Response to Training (HRT) and eight Low Response to Training (LRT) mature male rats. Fear conditioning, extinction, and extinction recall were performed as previously published (experiment 3 from Knox et al. [2012]). All fear conditioning, extinction, and extinction recall experiments were performed in four identical Coulbourn Instruments Rat Test Cages (12"W × 10"D × 12"H) (Whitehall, PA) containing a Shock Floor with 18 current‐carrying metal bars, a wall‐mounted speaker and in‐chamber lighting. Test cages were housed in wooden sound‐attenuating boxes. Tones were delivered via speakers mounted in the housing of the test cages and controlled by FreezeFrame data acquisition software (Coulbourn Instruments). Shocks were delivered through precision animal shockers (Coulbourn Instruments) also controlled by FreezeFrame software. Ceiling mounted cameras recorded behavior for analysis and FreezeFrame was used to assess freezing levels.

As previously published (Knox et al., 2012), two unique contexts were created using two different sets of olfactory and visual cues. Context A consists of 50 ml of 1% acetic acid solution placed in a small dish above the test cage and standard lighting which illuminates the chamber walls of the Rat Test Cages. Context B consists of 50 ml of a 1% ammonium hydroxide solution placed in a small dish above the test cage along with patterned paper placed on the chamber walls to alter the visual context. Other labs, as well as our own, have used these specific methods repeatedly in the past and found no evidence of increased stress with these concentrations of acetic acid or ammonium hydroxide, that is, they show no effects on behavior or HPA response (Knox et al., 2012).

Fear conditioned animals were exposed to five, 1 mA, 1‐s foot‐shocks paired with the cessation of a 10 s 80 dB tone in Context A. The first tone was presented 180 s after the animal was placed in the test cage and the subsequent tones occurred with a 60‐s inter‐tone interval. Sixty seconds after the last tone, animals were removed to their home cages. Fear extinction was conducted 24 hr after fear conditioning and was performed in the distinctly different Context B. Fear extinction consisted of 180 s acclimation to the new context and presentation of 30 ten‐second tones without the paired foot‐shock with each tone followed by a 60‐s inter‐tone interval. Extinction recall was assessed 24 hr after extinction and consisted of the animals being placed back into the same fear extinction context (Context B) for 180 s acclimation followed by 10 tones (60‐s inter‐tone interval), again without foot shock. The percent time spent immobile (freezing) within each 70‐s long block (the 10‐s tone and 60‐s inter‐tone interval combined) was assessed by setting threshold values of movement (number of pixels which moved between frames) via FreezeFrame Software. Threshold values were verified to be similar to experimenter‐confirmed immobility times.

### 2.3 Nociceptive testing

Rats (*n* = 8 HRT and 8 LRT) were conditioned to handling and to the IITC Model 336 T Paw Stimulator Analgesia Meter with a temperature‐controlled glass floor (IITC Model 400 Heated Base; IITC Life Science, CA, USA) for one week prior to nociceptive testing. Rats were allowed 30 min to habituate to the experiment chambers before nociceptive testing began. Nociceptive measures started at 2:00 PM and were conducted by an investigator blinded to the rat strain. Thermal hyperalgesia was assessed using the Hargreaves’ paw withdrawal latency (PWL) method (Hargreaves et al., 1988). As described in detail previously (Vanini et al., 2014), to test for thermal nociception a light beam was aimed directly at the plantar surface of a hind paw. The light was then switched from idle to the active intensity and a timer was simultaneously started at the onset of the stimulus. An automatic cut‐off time for the thermal stimulus was set at 15 s to prevent tissue damage. Upon withdrawal of the paw away from the thermal source, the light beam and the timer were stopped and the latency to withdrawal in seconds was recorded. Four withdrawal latency values were obtained from each paw by alternating the stimulus between the hind paws. A minimum of 30 s was allowed between thermal stimuli. No significant difference in PWL was observed between the left and right paw. The mean PWL from each rat was averaged to compare nociceptive thresholds between HRT and LRT rats.

### 2.4 Acute stress

Animals (4 LRT and 4 HRT) were restrained in custom‐built Plexiglas restraining devices for 2 hr beginning at ZT0. At ZT 2, animals were briefly anesthetized in a glass bell jar containing vaporized isoflurane. Biological tissues (blood, brain) were collected in under 2 min and quickly frozen and stored in a −80 freezer for later processing.

### 2.5 Western blot

Frozen brains were sectioned in 100 μm on a −20°C, Leica (Buffalo Grove, IL) cryostat and specific brain areas (amygdala, hippocampus, and locus coeruleus [LC]) were dissected into cold micro‐centrifuge tubes using a metal 2 mm circular tissue biopsy punch. Tissues from individual animals were homogenized in 300 μl of Homogenization Buffer (200 mM Tris, 10 mM EDTA, 10 mM Na_4_P_2_O_7_, 100 mM PMSF, 1x Sigma Phosphatase inhibitor II, III [Sigma Aldrich, St Louis, MO]). Total protein was quantified using BC Assay (Bio‐Rad, Hercules, CA) and all samples were equalized to the same concentration in Homogenization Buffer. Samples were combined with Laemmli Sample Buffer (Bio‐Rad), heated to 100°C for 5 min and then centrifuged at max speed for 3 min and loaded (35 μl) on a gel (4–15% TGX [Bio‐Rad, Hercules, CA]). Gel was electrophoresed at 100v for 60 min and then transferred to PVDF membrane at 4°C on ice at 100v for 1.5 hr. Blots were probed with mouse anti‐ppERK, 1:1000 (Sigma Aldrich), rabbit anti‐total ERK antibodies 1:1000 (Sigma Aldrich), rabbit anti‐GR, 1:1000 (Santa Cruz Biotechnology, Dallas, TX), rabbit anti‐BDNF antibodies, 1:1000 (Santa Cruz Biotechnology). Blots were visualized using a Li‐cor infrared detector and quantified using ImageJ software (NIH).

### 2.6 Stress hormone assay

Trunk blood was collected rapidly after decapitation and allowed to coagulate at room temperature for 30 min before being centrifuged at 1,000 *g* for 10 min. Serum was collected into clean micro‐centrifuge tubes and rat stress hormone levels were detected using the MILLIPLEX Rat Stress Hormone Magnetic Bead Panel (Millipore, St. Charles, MO.) according to the manufacturer's directions.

### 2.7 Immunostaining

Animals were sacrificed at ZT2, immediately following a 2‐hr physical restraint stressor. Four percent Paraformaldehyde fixed and frozen brain tissue was sectioned in 30 µm increments through the LC on a Leica (Buffalo Grove, IL) cryostat, stored in 1x PBS with sodium azide, and later processed for immunohistochemistry. Briefly, all sections were washed 3x for 10 min in 1x PBS, permeabilized in PBS–Triton (0.5%), blocked in 5% normal goat serum (in PBS–Triton 0.5%), and incubated in primary antibody overnight (24 hr) at 4°C. Sections were then washed in PBS and incubated in secondary antibody (24 hr) at 4°C. Tissue was mounted on poly‐lysine coated microscope glass and coverslipped under Prolong Gold (Molecular Probes). The following antibodies were used: mouse anti‐TH (ImmunoStar Hudson, WI), rabbit anti‐GR (Santa Cruz, Dallas, TX), and Alexa 488 conjugated anti‐mouse and Alexa 594 conjugated anti‐rabbit IgG (Molecular Probes) at 1:1000. Fluorescent images were collected on an Olympus fluorescent microscope provided by the University of Michigan Microscopy and Imaging core facility (University of Michigan). Immuno‐positive GR cells were counted using the ImageJ binary thresholding algorithm (San Diego Plugin). The average number of cells was compared between the LRT and HRT animals. The average values for each group were then evaluated using an independent sample t‐test.

### 2.8 Statistical analysis

Statistical comparison of time spent freezing on the fear conditioning, extinction, and extinction recall tasks was made between HRT and LRT selected rat strains using a 2‐way (time × group) ANOVA. Average freezing levels between groups were analyzed independently on fear conditioning, extinction, and extinction recall days.

Western Blot data were quantified using ImageJ software (NIH), normalized to control protein levels and statistical comparisons were made using GraphPad Prism Software.

One‐tailed and two‐tailed Mann–Whitney tests were performed on Western Blot, pain sensitization PWL, and hormone assay data where appropriate.

## 3 RESULTS

### 3.1 Fear extinction and recall is elevated in HRT animals

**FIGURE 1:**
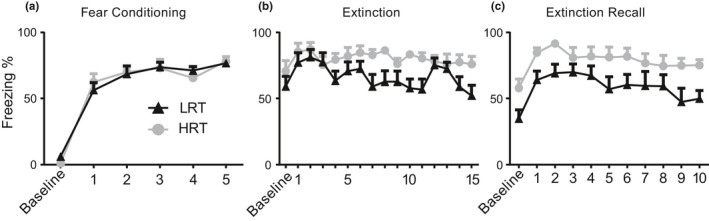
Extinction and Extinction Recall Deficits. On 3 successive days, we performed fear conditioning, extinction, and extinction recall. Freezing values (*y*‐axis) are plotted for the LRT (black triangles) and HRT (grey circles) rats against tones delivered (*x*‐axis). Fear conditioning consisted of one 3‐min baseline period followed by the presentation of 5, 1 mA foot shock presented at the cessation of a 10‐s 80 dB tone, in Context A (60‐s inter‐tone interval) (a). Fear extinction followed 24 hr after fear conditioning. All animals received 30 tones in Context B, on the same inter‐tone interval (15 tones shown). HRT rats froze significantly more than their LRT comparators (b). Fear extinction recall followed 48 hr after fear conditioning. All animals received 10 tones in the now extinguished Context B, on the same 60‐s inter‐tone interval. Again, HRT rats froze significantly more than their LRT comparators (c)

In order to test for differences in fear‐associated memory between LRT and HRT rats, we performed a 3‐day fear conditioning study (detailed in Methods). ANOVA of % freezing during the fear conditioning session (Day 1, Figure 1a) revealed a significant main effect of time (*F*_(5,100)_ = 87.3, *p* < 0.0001). There was no significant effect of genotype (*F*_(1,20)_ = 0.011, *p* = 0.92) or genotype × time interaction (*F*_(5,100)_ = 0.567, *p* = 0.73) (Figure 1a). ANOVA of cued freezing during fear extinction (Day2, Figure 1b) revealed a significant effect of genotype (*F*_(1,20)_ = 4.60, *p* = 0.044). There was no significant effect of time (*F*_(30,600)_ = 1.36, *p* = 0.096) or genotype × time interaction (*F*_(30,600)_ = 1.32, *p* = 0.124) (Figure 1b). ANOVA of cued response during fear extinction recall (Day3, Figure 1c) revealed significant main effects of genotype (*F*_(1,20)_ = 7.29, *p* = 0.014) and time (*F*_(10,200)_ = 6.11, *p* < 0.0001). There was no significant genotype × time interaction (*F*_(10,200)_ = 0.47, *p* = 0.91) (Figure 1c). The lack of extinction and recall shown in the HRT animals (Figure 1b,c, respectively) suggests that HRT rats have diminished the processing of fear‐associated memories compared to LRT rats.

### 3.2 Thermal nociception is not different between HRT and LRT rats

**FIGURE 2:**
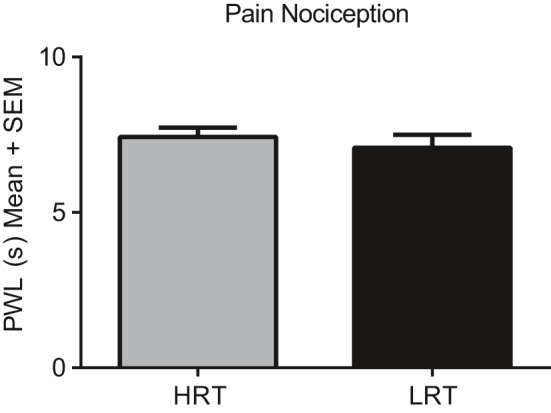
Thermal nociception. Paw withdrawal latency was measured using the IITC Model 336 T Paw Stimulator Analgesia Meter and found not to be statistically different between LRT and HRT rats

To test whether diminished fear extinction and recall may have been due to heightened pain sensitivity in HRT animals, we exposed eight HRT and eight LRT rats to thermal nociception testing as described in the methods section. A one‐tailed Mann–Whitney test revealed no significant differences in PWL to nociceptive thermal stimuli between HRT (*M* = 7.43 ± 0.83 s) and LRT (*M* = 7.08 ± 1.16 s) (*p* = 0.25) rats (Figure 2).

### 3.3 HRT and LRT rats display a differential hormonal response to acute stress

**FIGURE 3:**
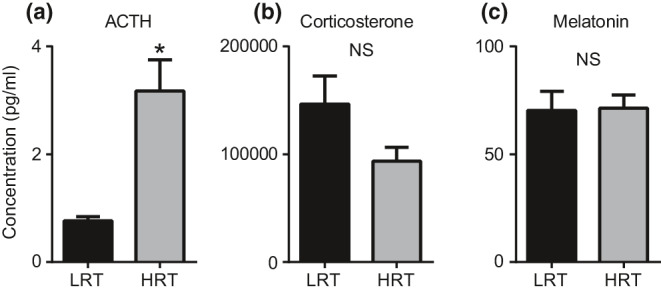
Stress hormone activation. The hormones ACTH, corticosterone, and melatonin were quantified after a brief 2‐hr physical restraint stress. Compared to LRT rats, HRT rats displayed a significant increase in ACTH (a). A non‐significant reduction in Corticosterone levels was found in HRT rats relative to the LRT rats (b). Melatonin activation was not different between LRT and HRT rats (c)

The exaggerated freezing responses in HRT rats on extinction and fear recall testing days suggest that HRT rats may have an altered hormonal response to stress. To test this hypothesis, we subjected four HRT and four LRT animals to 2 hr of physical restraint stress and immediately anesthetized, decapitated, and collected trunk blood for serum stress hormone detection. Serum ACTH, Corticosterone, and Melatonin levels were measured. Serum ACTH levels were significantly elevated in HRT rats (*M* = 3.17 ± 2.0 pg/ml) compared to LRT rats (*M* = 0.76 ± 0.19 pg/ml) (*p* = 0.0001) (Figure 3a). Serum Corticosterone (*p* = 0.21) and Melatonin (*p* = 0.94) levels were not significantly different between HRT and LRT rats (Figure 3b,c).

### 3.4 Hippocampal ERK activation

**FIGURE 4:**
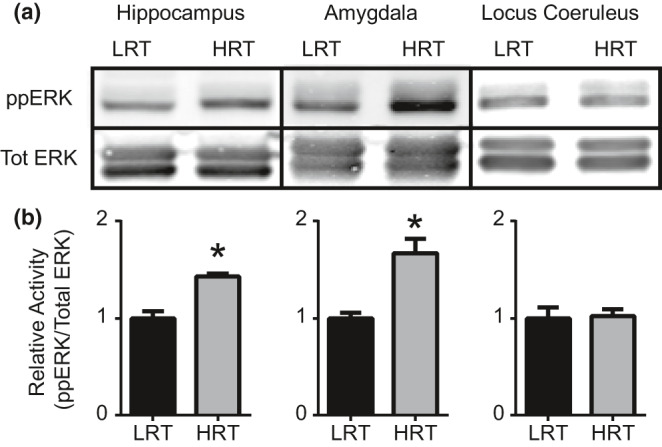
Extracellular signal‐regulated kinase (ERK) activation. Western blot was performed to quantify ERK activation following 2 hr of physical restraint. Western blots for pERK and Total ERK (Tot ERK) from hippocampus, amygdala, and LC tissues (a). Quantification was performed using ImageJ software and statistically significant differences in pERK/TotERK levels are shown in (b) (*p‐value <0.05)

**FIGURE 5:**
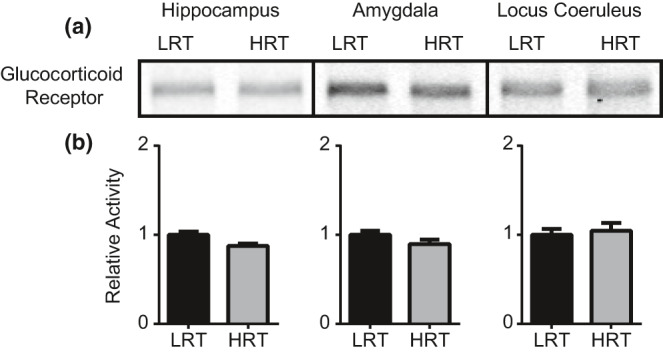
Glucocorticoid receptor. Western blot was performed to quantify GR levels following 2 hr of physical restraint. Western blots for GR from hippocampus, amygdala, and LC tissues (a). Quantification was performed using ImageJ software. No statistically significant differences were found for GR between LRT and HRT rats (b)

**FIGURE 6:**
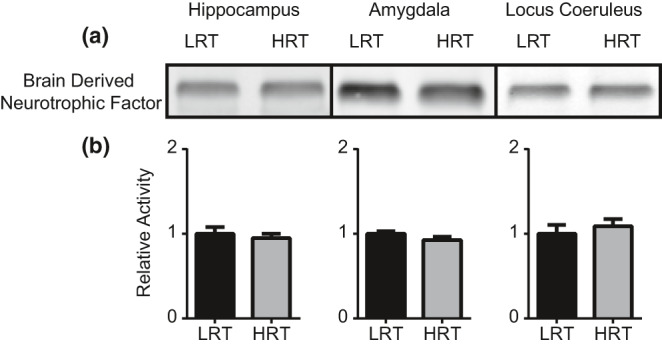
Brain‐derived neurotrophic factor. Western blot was performed to quantify BDNF levels following 2 hr of physical restraint. Western blots for BDNF from hippocampus, amygdala, and LC tissues (a). No statistically significant differences in BDNF levels were found between LRT and HRT rats (b)

Following blood collection for the assessment of hormonal changes in response to stress, we also collected brain tissue of neural circuits involved in fear memory processing (hippocampus, amygdala, and LC) and using Western blot, assessed changes in the plasticity‐related molecules pERK, GR, and BDNF. Two‐tailed Mann–Whitney tests revealed significant differences in hippocampal pERK activation between LRT (*M* = 1.0 ± 0.144) and HRT (*M* = 1.43 ± 0.059) (*p* = 0.028) animals (Figure 4). Amygdala pERK activation was also significantly different between LRT (*M* = 1.0 ± 0.12) and HRT (*M* = 1.67 ± 0.29) (*p* = 0.029) rats (Figure 4). pERK activation was not statistically different in the LC between LRT (*M* = 1.0 ± 0.22) and HRT (*M* = 1.02 ± 0.144) (*p* = 0.99) animals (Figure 4)a–c. No statistically significant differences were found in GR levels in the hippocampus, amygdala, or LC between LRT and HRT rats (*p* = 0.06, 0.11, and 0.65, respectively) (Figure 5). No statistically significant differences were found in BDNF levels in the hippocampus, amygdala, or LC between LRT and HRT rats (*p* = 0.99, 0.20, and 0.49, respectively) (Figure 6).

### 3.5 Differences in molecular and structural characteristics of the Locus Coeruleus

**FIGURE 7:**
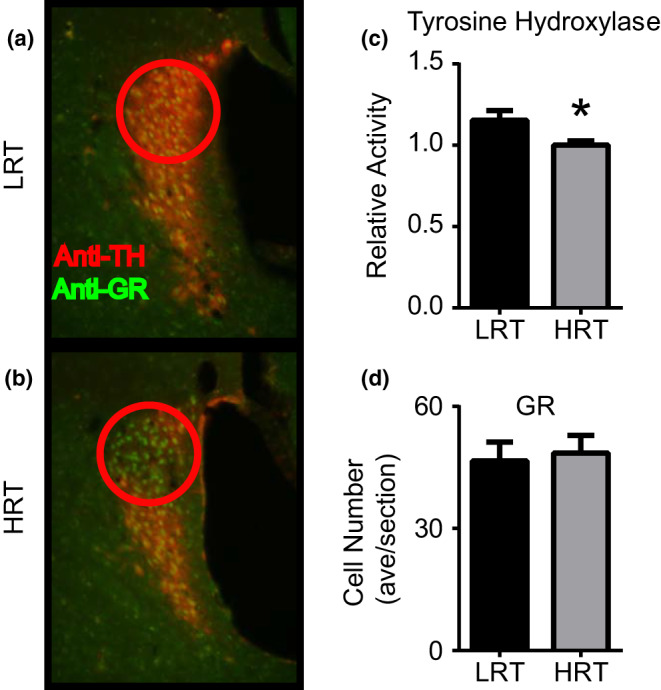
HRT rats have reduced tyrosine hydroxylase levels. Immuno‐histochemical analysis revealed that TH positive cells in the LC were significantly reduced in the HRT rats (b) compared to the LRT (a) rats. Histochemical analysis was verified using Western blot, confirming that TH levels were quantifiably different between LRT and HRT rats (c). This reduction in TH was not due to a reduction in cell numbers in the LC, the number of GR containing cells in the LC remained the same between LRT and HRT animals (d). (*p‐value <0.05)

Based on the data shown here and previously published research on stress, we hypothesized that the noradrenergic LC may differentially undergo molecular (Li et al., 2011; Weiss et al., 1994) or structural changes (Bracha et al., 2005) between LRT and HRT rats in response to stress. Immunohistochemistry for TH, which specifically stains the noradrenergic cells of the LC, revealed a differential anatomical localization of TH containing cells between the LRT and HRT rats. The dorsal LC lacked staining of TH in the HRT animals that are present in LRT animals and normally present in wild‐type animals (Circles in Figure 7a,b), while the dorsal section of the LC looked relatively similar between groups. Using Western Blot, we further quantified the change in TH between the LRT and HRT animals. A one‐tailed Mann–Whitney test revealed significant differences in TH levels between HRT (*M* = 1.00 ± 0.04) and LRT (*M* = 1.15 ± 0.12) (*p* = 0.028) rats (Figure 7c). We additionally confirmed that this change in TH was not due to differences in cell number within the LC, as the response to physical restraint resulted in the same average number of GR positive cells between HRT (*M* = 48.49 ± 4.3) and LRT (*M* = 46.57 ± 4.5) (*p* = 0.65) rats (Figure 7d).

## 4 DISCUSSION

Physical fitness has been hypothesized to be a resilience factor to stress exposure. Additionally, physical activity has the potential to assist in the treatment of stress‐related and anxiety disorders (Taylor et al., 2008). Yet, a mechanistic understanding of the association between exercise activity and stress is lacking. The response to exercise training is noted as having wide heterogeneity such that some individuals improve exercise capacity more than others (e.g., higher rise in maximal oxygen consumption) (Vollaard et al., 2009), largely due to unidentified genotype‐environment interactions (Bouchard et al., 2012). This work is the first of its kind to identify molecular and anatomical pathways that overlap in their function for stress regulation and adaptation to exercise.

In this work, we have shown that, compared to LRT rats, HRT rats show impaired fear‐associated memory processing following fear conditioning. HRT rats have been artificially selected to show an enhanced ability to respond to aerobic training. However, the selection process consisting of repeated exposure to forced treadmill exercise at increasing speed and duration, designed to select animals’ responsiveness to running training, may have additionally co‐selected traits for heightened arousal and stress sensitivity. Exercise training by these methods modulates the dose–response phenomenon that balances the health‐promoting effects of exercise with the exercise‐induced production of oxidative stress markers (Radak et al., 2017) Indeed, aberrant ACTH signaling arises as a consequence of exposure to stress (Knox et al., 2012). Further, similar to the HRT animals shown here, ERK activation has been shown to be increased in the amygdala when presented with extreme stress (Liu et al., 2010; Xiao et al., 2011). Therefore, the selection process for exercise responsiveness has generated a contrasting animal system that has molecular and behavioral characteristics similar to animals more (HRT) and less responsive (LRT) to extreme stress.

The HRT and LRT rats showed no differences in nociception (Figure 2). Therefore, the behavioral learning differences detected during fear extinction and fear extinction recall cannot be attributed to an increased perception of pain during fear conditioning. More than likely, these fear‐associated memory variances are due to molecular signaling or anatomical remodeling abnormalities. Indeed, pERK activation was greater in HRT rats in the hippocampus and the amygdala following 2 hours of physical restraint stress (Figure 4), and the LC showed significant histochemical changes which may contribute to behavioral differences in response to this fear conditioning paradigm.

Increased noradrenergic signaling contributes to features of hyper‐arousal such as those seen in HRT rats with reduced abilities to extinguish fear‐associated memories (Geracioti et al., 2001; Mellman et al., 1995; Southwick et al., 1993, 1999; Strawn & Geracioti, 2008). To examine the contribution of the noradrenergic LC in mediating this behavior, we exposed animals to a 2‐hr physical restraint stress and assessed differences in GR and TH, a rate‐limiting step for catecholamine synthesis. The structural abnormalities in TH localization in the LC revealed a reduced number of LC cells and suggest that some of the behavioral impairments may be due, in part, to structural abnormalities in the noradrenergic LC. Loss of noradrenergic cells is also seen in human populations exposed to extreme levels of physical or psychological stress (Bracha et al., 2005). These data further connect the stress response system to exercise physiology.

**FIGURE 8:**
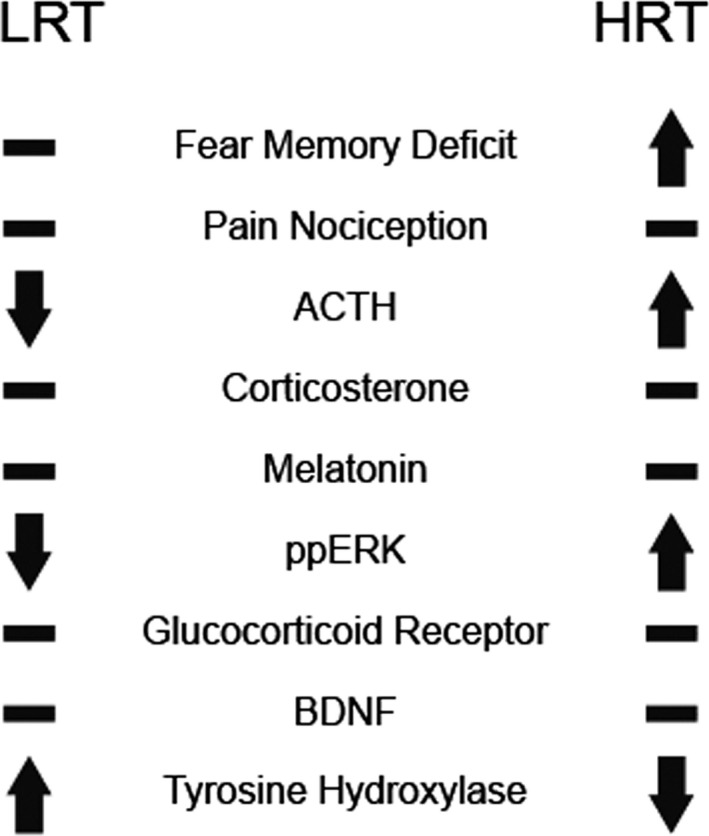
Summary of differences between LRT and HRT animals. LRT and HRT animals show unique biochemical and behavioral measures in response to stress and exposure to a fear‐associated memory test

Acute stress can provide useful information on how the brain responds to over‐activity of some molecular systems and underactivity of others (summarized in Figure 8). This study provides a framework by which the molecular response to acute stress can be examined within the context of exercise. These data, showing that ERK activation is unique between HRT and LRT animals, identify a unique molecular pathway underlying stress and exercise neurobiology. Future work may focus on the contribution of ERK activation in physical exercise as a potential intervention for stress‐induced maladaptive behaviors such as fear‐memory impairments and hyper‐arousal. Additional examination of the BDNF pathway is also warranted given that exercise is sufficient to activate BDNF in the brains of LRT animals exposed to exercise, but not in HRT animals (Marton et al., 2016).

As a first report, the data presented here show that rats selectively bred for high adaptation for exercise training have a higher level of molecular and structural brain adaptations in response to stress and a higher level of retention for fear‐related memory compared to those rats selected for a lessened adaptation. Further work is required to assess if the HRT‐specific fear‐associated memory impairments are the result of increased threat avoidance behavior or increased threat vigilance (Waters & Kershaw, 2015) and to understand the mechanisms regulating impaired fear‐associated memory processing in these animals in a more biologically comprehensive cross‐sectional and longitudinal studies. This study was uniquely conducted in aged animals which lend itself to further efforts needed to assess the association between stress disorders and impaired cognitive performance (Yaffe et al., 2010).

Last, exercise and stress activate many similar anatomical and molecular pathways. For example, both exercise (Jacks et al., 2002) and stress (McEwen, 2000) have been shown to increase cortisol response via the ACTH stimulus. Further complicating the story is the evidence that exercise type, intensity, and duration along with genetic predisposition for high capacity for aerobic training is critical to the beneficial response in the brain for adult hippocampal neurogenesis (Nokia et al., 2016). There is also the idea that the practice of extreme sports with high adrenaline and cortisol release such as whitewater rafting, rock climbing, and skydiving may in turn help to replace bad memories previously linked with high adrenaline and cortisol levels in combat veterans (xsports4vets.org). Indeed, physical activity is prescribed to help reduce symptoms of post‐traumatic stress disorder (PTSD) and promote recovery and resilience. However, a non‐linear and U‐shaped association showing veterans reporting no weekly exercise and daily exercise having the highest prevalence of probable PTSD is consistent with endpoints of the exercise‐associated hormesis curve (Adams et al., 2020; Radak et al., 2008). The importance of exercise capacity as a biomarker in the onset of stress‐related disorders is currently unknown and the mechanisms of overlapping stress hormone molecular pathways are still poorly understood (McEwen, 2015). It is our contention, therefore, that the HRT and LRT rats we developed may provide a differential genetic substrate to aid in our understanding of the complex biological process by which stress and physical exercise interact.

## CONFLICT OF INTEREST

The authors have no conflict of interest to declare.

## References

[phy214716-bib-0001] Adams, T. G. , Forte, J. , Fogle, B. M. , Southwick, S. M. , & Pietrzak, R. H. (2020). Self‐reported exercise frequency and PTSD: Results from the National Health and Resilience in Veterans Study. Acta Psychiatrica Scandinavica, 142(6), 486–495. 10.1111/acps.13234 32961606

[phy214716-bib-0002] Ahmadiasl, N. , Alaei, H. , & Hanninen, O. (2003). Effect of exercise on learning, memory and levels of epinephrine in rats’ hippocampus. Journal of Sports Science & Medicine, 2, 106–109. 24627662 PMC3942636

[phy214716-bib-0003] Baker, D. G. , West, S. A. , Nicholson, W. E. , Ekhator, N. N. , Kasckow, J. W. , Hill, K. K. , Bruce, A. B. , Orth, D. N. , & Geracioti, T. D. Jr (1999). Serial CSF corticotropin‐releasing hormone levels and adrenocortical activity in combat veterans with posttraumatic stress disorder. American Journal of Psychiatry, 156, 585–588. 10.1176/ajp.156.4.585 10200738

[phy214716-bib-0004] Barbour, K. A. , Edenfield, T. M. , & Blumenthal, J. A. (2007). Exercise as a treatment for depression and other psychiatric disorders: A review. Journal of Cardiopulmonary Rehabilitation and Prevention, 27, 359–367. 18197069 10.1097/01.HCR.0000300262.69645.95

[phy214716-bib-0005] Blumenthal, J. A. , Babyak, M. A. , Doraiswamy, P. M. , Watkins, L. , Hoffman, B. M. , Barbour, K. A. , Herman, S. , Craighead, W. E. , Brosse, A. L. , Waugh, R. , Hinderliter, A. , & Sherwood, A. (2007). Exercise and pharmacotherapy in the treatment of major depressive disorder. Psychosomatic Medicine, 69, 587–596. 10.1097/PSY.0b013e318148c19a 17846259 PMC2702700

[phy214716-bib-0006] Bouchard, C. , Blair, S. N. , Church, T. S. , Earnest, C. P. , Hagberg, J. M. , Häkkinen, K. , Jenkins, N. T. , Karavirta, L. , Kraus, W. E. , Leon, A. S. , Rao, D. C. , Sarzynski, M. A. , Skinner, J. S. , Slentz, C. A. , & Rankinen, T. (2012). Adverse metabolic response to regular exercise: Is it a rare or common occurrence? PLoS One, 7, e37887 10.1371/journal.pone.0037887 22666405 PMC3364277

[phy214716-bib-0007] Bracha, H. S. , Garcia‐Rill, E. , Mrak, R. E. , & Skinner, R. (2005). Postmortem locus coeruleus neuron count in three American veterans with probable or possible war‐related PTSD. Journal of Neuropsychiatry and Clinical Neurosciences, 17, 503–509. 10.1176/jnp.17.4.503 PMC4484762 16387990

[phy214716-bib-0008] Bremner, J. D. , Elzinga, B. , Schmahl, C. , & Vermetten, E. (2007). Structural and functional plasticity of the human brain in posttraumatic stress disorder. Progress in Brain Research, 167, 171–186. 10.1016/S0079-6123(07)67012-5 PMC3226705 18037014

[phy214716-bib-0009] Chandramohan, Y. , Droste, S. K. , Arthur, J. S. , & Reul, J. M. (2008). The forced swimming‐induced behavioural immobility response involves histone H3 phospho‐acetylation and c‐Fos induction in dentate gyrus granule neurons via activation of the N‐methyl‐D‐aspartate/extracellular signal‐regulated kinase/mitogen‐ and stress‐activated kinase signalling pathway. European Journal of Neuroscience, 27, 2701–2713. 10.1111/j.1460-9568.2008.06230.x 18513320

[phy214716-bib-0010] de Kloet, E. R. , Joels, M. , & Holsboer, F. (2005). Stress and the brain: From adaptation to disease. Nature Reviews Neuroscience, 6, 463–475. 15891777 10.1038/nrn1683

[phy214716-bib-0011] Denham, J. , Marques, F. , O’Brien, B. , & Charchar, F. (2014). Exercise: Putting action into our epigenome. Sports Medicine (Auckland, N. Z.), 44, 189–209. 10.1007/s40279-013-0114-1 24163284

[phy214716-bib-0012] Dillard, C. J. , Litov, R. E. , Savin, W. M. , Dumelin, E. E. , & Tappel, A. L. (1978). Effects of exercise, vitamin E, and ozone on pulmonary function and lipid peroxidation. Journal of Applied Physiology: Respiratory, Environmental and Exercise Physiology, 45, 927–932. 10.1152/jappl.1978.45.6.927 730598

[phy214716-bib-0013] Freund, B. J. , Shizuru, E. M. , Hashiro, G. M. , & Claybaugh, J. R. (1991). Hormonal, electrolyte, and renal responses to exercise are intensity dependent. Journal of Applied Physiology, 70, 900–906. 1827109 10.1152/jappl.1991.70.2.900

[phy214716-bib-0014] Garfinkel, S. N. , Abelson, J. L. , King, A. P. , Sripada, R. K. , Wang, X. , Gaines, L. M. , & Liberzon, I. (2014). Impaired contextual modulation of memories in PTSD: an fMRI and psychophysiological study of extinction retention and fear renewal. Journal of Neuroscience, 34, 13435–13443. 25274821 10.1523/JNEUROSCI.4287-13.2014 PMC4262698

[phy214716-bib-0015] Geracioti, T. D. Jr , Baker, D. G. , Ekhator, N. N. , West, S. A. , Hill, K. K. , Bruce, A. B. , Schmidt, D. , Rounds‐Kugler, B. , Yehuda, R. , Keck, P. E. Jr , & Kasckow, J. W. (2001). CSF norepinephrine concentrations in posttraumatic stress disorder. American Journal of Psychiatry, 158, 1227–1230. 10.1176/appi.ajp.158.8.1227 11481155

[phy214716-bib-0016] Hargreaves, K. , Dubner, R. , Brown, F. , Flores, C. , & Joris, J. (1988). A new and sensitive method for measuring thermal nociception in cutaneous hyperalgesia. Pain, 32, 77–88. 3340425 10.1016/0304-3959(88)90026-7

[phy214716-bib-0017] Holmes, P. V. (2014). Trophic mechanisms for exercise‐induced stress resilience: Potential role of interactions between BDNF and Galanin. Frontiers in Psychiatry, 5, 90. 25120496 10.3389/fpsyt.2014.00090 PMC4112800

[phy214716-bib-0018] Jacks, D. E. , Sowash, J. , Anning, J. , McGloughlin, T. , & Andres, F. (2002). Effect of exercise at three exercise intensities on salivary cortisol. Journal of Strength and Conditioning Research, 16(2), 286–289. 10.1519/00124278-200205000-00018 11991783

[phy214716-bib-0019] Knox, D. , Nault, T. , Henderson, C. , & Liberzon, I. (2012). Glucocorticoid receptors and extinction retention deficits in the single prolonged stress model. Neuroscience, 223, 163–173. 10.1016/j.neuroscience.2012.07.047 22863672

[phy214716-bib-0020] Koch, L. G. , Pollott, G. E. , & Britton, S. L. (2013). Selectively bred rat model system for low and high response to exercise training. Physiological Genomics, 45, 606–614. 23715262 10.1152/physiolgenomics.00021.2013 PMC3727016

[phy214716-bib-0021] Lessard, S. J. , Rivas, D. A. , Alves‐Wagner, A. B. , Hirshman, M. F. , Gallagher, I. J. , Constantin‐Teodosiu, D. , Atkins, R. , Greenhaff, P. L. , Qi, N. R. , Gustafsson, T. , Fielding, R. A. , Timmons, J. A. , Britton, S. L. , Koch, L. G. , & Goodyear, L. J. (2013). Resistance to aerobic exercise training causes metabolic dysfunction and reveals novel exercise‐regulated signaling networks. Diabetes, 62, 2717–2727. 10.2337/db13-0062 23610057 PMC3717870

[phy214716-bib-0022] Li, M. , Han, F. , & Shi, Y. (2011). Expression of locus coeruleus mineralocorticoid receptor and glucocorticoid receptor in rats under single‐prolonged stress. Neurological Sciences, 32(4), 625–631. 10.1007/s10072-011-0597-1 21584742

[phy214716-bib-0023] Liberzon, I. , & Sripada, C. S. (2008). The functional neuroanatomy of PTSD: a critical review. Progress in Brain Research, 167, 151–169. 18037013 10.1016/S0079-6123(07)67011-3

[phy214716-bib-0024] Liu, H. , Li, H. , Xu, A. , Kan, Q. , & Liu, B. (2010). Role of phosphorylated ERK in amygdala neuronal apoptosis in single‐prolonged stress rats. Molecular Medicine Reports, 3, 1059–1063. 10.3892/mmr.2010.362 21472355

[phy214716-bib-0025] Maes, M. , Lin, A. H. , Delmeire, L. , Van Gastel, A. , Kenis, G. , De Jongh, R. , & Bosmans, E. (1999). Elevated serum interleukin‐6 (IL‐6) and IL‐6 receptor concentrations in posttraumatic stress disorder following accidental man‐made traumatic events. Biological Psychiatry, 45, 833–839. 10.1016/S0006-3223(98)00131-0 10202570

[phy214716-bib-0026] Martini, C. , Da Pozzo, E. , Carmassi, C. , Cuboni, S. , Trincavelli, M. L. , Massimetti, G. , Marazziti, D. , & Dell'Osso, L. (2013). Cyclic adenosine monophosphate responsive element binding protein in post‐traumatic stress disorder. The World Journal of Biological Psychiatry, 14(5), 396–402. 10.3109/15622975.2011.577189 21696331

[phy214716-bib-0027] Marton, O. , Koltai, E. , Takeda, M. , Koch, L. G. , Britton, S. L. , Davies, K. J. , Boldogh, I. , & Radak, Z. (2014). Mitochondrial biogenesis‐associated factors underlie the magnitude of response to aerobic endurance training in rats. Pflügers Archiv ‐ European Journal of Physiology, 467(4), 779–788. 10.1007/s00424-014-1554-7 24943897 PMC4272336

[phy214716-bib-0028] Marton, O. , Koltai, E. , Takeda, M. , Mimura, T. , Pajk, M. , Abraham, D. , Koch, L. G. , Britton, S. L. , Higuchi, M. , Boldogh, I. , & Radak, Z. (2016). The rate of training response to aerobic exercise affects brain function of rats. Neurochemistry International, 99, 16–23. 10.1016/j.neuint.2016.05.012 27262284

[phy214716-bib-0029] McEwen, B. S. (2000). The neurobiology of stress: from serendipity to clinical relevance. Brain Research, 886, 172–189. 11119695 10.1016/s0006-8993(00)02950-4

[phy214716-bib-0030] McEwen, B. S. (2015). Biomarkers for assessing population and individual health and disease related to stress and adaptation. Metabolism, 64, S2–S10. 10.1016/j.metabol.2014.10.029 25496803

[phy214716-bib-0031] Mellman, T. A. , Kumar, A. , Kulick‐Bell, R. , Kumar, M. , & Nolan, B. (1995). Nocturnal/daytime urine noradrenergic measures and sleep in combat‐related PTSD. Biological Psychiatry, 38, 174–179. 7578660 10.1016/0006-3223(94)00238-X

[phy214716-bib-0032] Nokia, M. S. , Lensu, S. , Ahtiainen, J. P. , Johansson, P. P. , Koch, L. G. , Britton, S. L. , & Kainulainen, H. (2016). Physical exercise increases adult hippocampal neurogenesis in male rats provided it is aerobic and sustained. The Journal of Physiology, 594, 1855–1873. 26844666 10.1113/JP271552 PMC4818598

[phy214716-bib-0033] Pitman, R. K. , Rasmusson, A. M. , Koenen, K. C. , Shin, L. M. , Orr, S. P. , Gilbertson, M. W. , Milad, M. R. , & Liberzon, I. (2012). Biological studies of post‐traumatic stress disorder. Nature Reviews Neuroscience, 13, 769–787. 23047775 10.1038/nrn3339 PMC4951157

[phy214716-bib-0034] Radak, Z. , Chung, H. Y. , Koltai, E. , Taylor, A. W. , & Goto, S. (2008). Exercise, oxidative stress and hormesis. Ageing Research Reviews, 7, 34–42. 17869589 10.1016/j.arr.2007.04.004

[phy214716-bib-0035] Radak, Z. , Ishihara, K. , Tekus, E. , Varga, C. , Posa, A. , Balogh, L. , Boldogh, I. , & Koltai, E. (2017). Exercise, oxidants, and antioxidants change the shape of the bell‐shaped hormesis curve. Redox Biology, 12, 285–290. 28285189 10.1016/j.redox.2017.02.015 PMC5345970

[phy214716-bib-0036] Rothman, S. M. , & Mattson, M. P. (2013). Activity‐dependent, stress‐responsive BDNF signaling and the quest for optimal brain health and resilience throughout the lifespan. Neuroscience, 239, 228–240. 23079624 10.1016/j.neuroscience.2012.10.014 PMC3629379

[phy214716-bib-0037] Sciolino, N. R. , & Holmes, P. V. (2012). Exercise offers anxiolytic potential: a role for stress and brain noradrenergic‐galaninergic mechanisms. Neuroscience and Biobehavioral Reviews, 36, 1965–1984. 22771334 10.1016/j.neubiorev.2012.06.005 PMC4815919

[phy214716-bib-0038] Southwick, S. M. , Bremner, J. D. , Rasmusson, A. , Morgan, C. A. 3rd , Arnsten, A. , & Charney, D. S. (1999). Role of norepinephrine in the pathophysiology and treatment of posttraumatic stress disorder. Biological Psychiatry, 46, 1192–1204. 10560025 10.1016/s0006-3223(99)00219-x

[phy214716-bib-0039] Southwick, S. M. , Krystal, J. H. , Morgan, C. A. , Johnson, D. , Nagy, L. M. , Nicolaou, A. , Heninger, G. R. , & Charney, D. S. (1993). Abnormal noradrenergic function in posttraumatic stress disorder. Archives of General Psychiatry, 50, 266–274. 8466387 10.1001/archpsyc.1993.01820160036003

[phy214716-bib-0040] Spivak, B. , Shohat, B. , Mester, R. , Avraham, S. , Gil‐Ad, I. , Bleich, A. , Valevski, A. , & Weizman, A. (1997). Elevated levels of serum interleukin‐1 beta in combat‐related posttraumatic stress disorder. Biological Psychiatry, 42, 345–348. 9276074 10.1016/S0006-3223(96)00375-7

[phy214716-bib-0041] Sripada, R. K. , King, A. P. , Welsh, R. C. , Garfinkel, S. N. , Wang, X. , Sripada, C. S. , & Liberzon, I. (2012). Neural dysregulation in posttraumatic stress disorder: evidence for disrupted equilibrium between salience and default mode brain networks. Psychosomatic Medicine, 74, 904–911. 23115342 10.1097/PSY.0b013e318273bf33 PMC3498527

[phy214716-bib-0042] Strawn, J. R. , & Geracioti, T. D. Jr (2008). Noradrenergic dysfunction and the psychopharmacology of posttraumatic stress disorder. Depress Anxiety, 25, 260–271. 10.1002/da.20292 17354267

[phy214716-bib-0043] Takei, S. , Morinobu, S. , Yamamoto, S. , Fuchikami, M. , Matsumoto, T. , & Yamawaki, S. (2011). Enhanced hippocampal BDNF/TrkB signaling in response to fear conditioning in an animal model of posttraumatic stress disorder. Journal of Psychiatric Research, 45, 460–468. 10.1016/j.jpsychires.2010.08.009 20863519

[phy214716-bib-0044] Taylor, M. K. , Markham, A. E. , Reis, J. P. , Padilla, G. A. , Potterat, E. G. , Drummond, S. P. , & Mujica‐Parodi, L. R. (2008). Physical fitness influences stress reactions to extreme military training. Military Medicine, 173, 738–742. 10.7205/MILMED.173.8.738 18751589

[phy214716-bib-0045] van Praag, H. , Christie, B. R. , Sejnowski, T. J. , & Gage, F. H. (1999). Running enhances neurogenesis, learning, and long‐term potentiation in mice. Proceedings of the National Academy of Sciences of the United States of America, 96, 13427–13431. 10.1073/pnas.96.23.13427 10557337 PMC23964

[phy214716-bib-0046] Vanderheyden, W. M. , Poe, G. R. , & Liberzon, I. (2014). Trauma exposure and sleep: using a rodent model to understand sleep function in PTSD. Experimental Brain Research, 232(5), 1575–1584. 10.1007/s00221-014-3890-4 24623353

[phy214716-bib-0047] Vanini, G. , Nemanis, K. , Baghdoyan, H. A. , & Lydic, R. (2014). GABAergic transmission in rat pontine reticular formation regulates the induction phase of anesthesia and modulates hyperalgesia caused by sleep deprivation. European Journal of Neuroscience, 40, 2264–2273. 10.1111/ejn.12571 PMC4107042 24674578

[phy214716-bib-0048] Vollaard, N. B. , Constantin‐Teodosiu, D. , Fredriksson, K. , Rooyackers, O. , Jansson, E. , Greenhaff, P. L. , Timmons, J. A. , & Sundberg, C. J. (2009). Systematic analysis of adaptations in aerobic capacity and submaximal energy metabolism provides a unique insight into determinants of human aerobic performance. Journal of Applied Physiology, 106, 1479–1486. 10.1152/japplphysiol.91453.2008 19196912

[phy214716-bib-0049] Waters, A. M. , & Kershaw, R. (2015). Direction of attention bias to threat relates to differences in fear acquisition and extinction in anxious children. Behavior Research and Therapy, 64, 56–65. 10.1016/j.brat.2014.11.010 25540863

[phy214716-bib-0050] Weiss, G. K. , Ratner, A. , Voltura, A. , Savage, D. , Lucero, K. , & Castillo, N. (1994). The effect of two different types of stress on locus coeruleus alpha‐2 receptor binding. Brain Research Bulletin, 33, 219–221. 10.1016/0361-9230(94)90255-0 7903904

[phy214716-bib-0051] Widegren, U. , Ryder, J. W. , & Zierath, J. R. (2001). Mitogen‐activated protein kinase signal transduction in skeletal muscle: effects of exercise and muscle contraction. Acta Physiologica Scandinavica, 172, 227–238. 11472310 10.1046/j.1365-201x.2001.00855.x

[phy214716-bib-0052] Wisloff, U. , Bye, A. , Stolen, T. , Kemi, O. J. , Pollott, G. E. , Pande, M. , McEachin, R. C. , Britton, S. L. , & Koch, L. G. (2015). Blunted cardiomyocyte remodeling response in exercise‐resistant rats. Journal of the American College of Cardiology, 65, 1378–1380. 10.1016/j.jacc.2015.01.041 25835453

[phy214716-bib-0053] Xiao, B. , Han, F. , Wang, H. T. , & Shi, Y. X. (2011). Single‐prolonged stress induces increased phosphorylation of extracellular signal‐regulated kinase in a rat model of post‐traumatic stress disorder. Molecular Medicine Reports, 4, 445–449. 21468590 10.3892/mmr.2011.459

[phy214716-bib-0054] Yaffe, K. , Vittinghoff, E. , Lindquist, K. , Barnes, D. , Covinsky, K. E. , Neylan, T. , Kluse, M. , & Marmar, C. (2010). Posttraumatic stress disorder and risk of dementia among US veterans. Archives of General Psychiatry, 67, 608–613. 10.1001/archgenpsychiatry.2010.61 20530010 PMC2933793

[phy214716-bib-0055] Yehuda, R. , Golier, J. A. , Halligan, S. L. , Meaney, M. , & Bierer, L. M. (2004). The ACTH response to dexamethasone in PTSD. American Journal of Psychiatry, 161, 1397–1403. 10.1176/appi.ajp.161.8.1397 15285965

[phy214716-bib-0056] Zen, A. L. , Whooley, M. A. , Zhao, S. , & Cohen, B. E. (2012). Post‐traumatic stress disorder is associated with poor health behaviors: Findings from the Heart and Soul Study In: American Geriatrics Society meeting, 2010; Preliminary data for this article was presented as an abstract at the aforementioned conference American Psychological Association, p. 194. 10.1037/a0025989 PMC3295904 22023435

[phy214716-bib-0057] Zovkic, I. B. , & Sweatt, J. D. (2013). Epigenetic mechanisms in learned fear: Implications for PTSD. Neuropsychopharmacology, 38, 77–93. 22692566 10.1038/npp.2012.79 PMC3521992

